# Synthesis of Ni_3_Si_4_O_10_(OH)_2_ Porous Microspheres as Support of Pd Catalyst for Hydrogenation Reaction

**DOI:** 10.3390/nano9070998

**Published:** 2019-07-11

**Authors:** Tingting Wang, Chenyuan Liu, Xinxin Ma, Wancheng Zhu, Xiaoxia Lv, Heng Zhang

**Affiliations:** School of Chemistry and Chemical Engineering, Qufu Normal University, Shandong 273165, China

**Keywords:** nickel phyllosilicate, hydrothermal synthesis, supported catalyst, hydrogenation

## Abstract

Nickel phyllosilicates have attracted much attention owing to their potential applications in various fields. Herein, Ni_3_Si_4_O_10_(OH)_2_ porous microspheres (NiSi-PMs) with a diameter of 1.2 to 3.2 μm were successfully fabricated via a urea-assisted hydrothermal method, and subsequently used to prepare supported Pd catalyst. Characterizations of the NiSi-PMs and the obtained catalyst, combined with the catalytic performance for the hydrogenation reaction, are presented and discussed. The BET surface area and pore volume of the NiSi-PMs were 196.2 m^2^ g^−1^ and 0.70 cm^3^ g^−1^, respectively. The Pd/NiSi-PMs catalyst exhibited remarkable catalytic activity for the hydrogenation of styrene under mild conditions, with a turnover frequency of 5234 h^−1^, and the catalyst was recovered and recycled for six consecutive cycles without any discernible loss of activity. H_2_-TPR and H_2_-TPD revealed that the activity of the catalysts was closely related to the adsorption property for hydrogen. The present Ni_3_Si_4_O_10_(OH)_2_ supported Pd catalyst afforded a promising and competitive candidate for heterogeneous catalysis.

## 1. Introduction

Noble metal catalysts have attracted considerable attention for their wide applications in various chemical reactions. Noble metals are usually dispersed finely on a support for cost savings, and more significantly, for the efficient use of the catalytic active sites. Generally, the solid supports upon which to disperse the noble metals need to have a large surface area, good structural stability, and the interaction between the support and the active phase has significant effects on the catalytic activity and the stability of the supported noble metals. Besides conventional supports, such as activated carbon, silica, and alumina, solid materials with a specific pore structure, such as ordered mesoporous materials [1,2,3], metal-organic frameworks (MOFs) [4,5], three dimensionally ordered macroporous (3DOM) materials [6,7], and other multifunctional composites/hybrids [8,9,10] are attracting increasing interest for the preparation of supported noble metal catalysts. Perovskite materials have also been explored for supporting noble metals and the so-called “smart” catalysts were developed, in which the noble metal can reversibly move into and out of the perovskite lattice with a change in the oxidizing and reducing environments [11,12,13]. This movement was found to suppress the growth of the noble metal particles in high-temperature reactions [13].

Among the various materials used for preparing supported metal catalyst, phyllosilicates as a big family of layered silicates, including different natural clays, such as montmorillonite, saponite, attapulgite, and many synthetic metal phyllosilicates, have been widely studied because of their specific structural features, ion exchange and adsorption properties, and the ease of modification through inorganic or organic functionalization. In recent years, increasing studies have been conducted on nickel phyllosilicates, which were used as the precursors to prepare nickel based catalysts [14,15,16,17,18]. The difference of the Ni/Si ratio generates two types of structures: 1:1 and 2:1 nickel phyllosilicates with the formula of Ni_3_Si_2_O_5_(OH)_4_ and Ni_3_Si_4_O_10_(OH)_2_. In the former, each layer consists of one tetrahedral SiO_4_ sheet and one octahedral NiO_6_ sheet. In the latter, two tetrahedral SiO_4_ sheets interleave with one octahedral NiO_6_ sheet like sandwiches. The preparation and application of Ni_3_Si_2_O_5_(OH)_4_ has obtained more concerns. Nanotubular Ni_3_Si_2_O_5_(OH)_4_ has been synthesized by hydrothermal methods in a strongly alkaline environment and obtained particular interest as lithium battery anode support materials [19,20,21]. Meanwhile, three dimensional hierarchical assemblies have attracted much attention owing to their potential value for applications, like catalysis, the treatment of waste water as well as lithium batteries. Many efforts have been made to synthesize Ni_3_Si_2_O_5_(OH)_4_ with a hollow sphere or core-shell structure. Usually, SiO_2_ microspheres are used as templates, on the surface of which Ni_3_Si_2_O_5_(OH)_4_ is formed through the reaction of nickel species and the SiO_2_ [22,23]. However, this preparation route often requires hard-controlled conditions and subsequent complicated procedures for the removal of templates. Moreover, the synthesis of the 2:1 nickel phyllosilicate Ni_3_Si_4_O_10_(OH)_2_ has rarely been reported in the literature. Herein, Ni_3_Si_4_O_10_(OH)_2_ porous microspheres, which were denoted as NiSi-PMs in the present work, have been synthesized by a simple hydrothermal method using NiCl_2_, tetraethoxysilane (TEOS), and urea as the raw materials. Furthermore, NiSi-PMs were used to prepare supported Pd catalyst, which exhibited a very high catalytic activity for the hydrogenation of styrene.

## 2. Materials and Methods

### 2.1. Synthesis of NiSi-PMs

All the chemicals were analytical grade and obtained from Sinopharm Chemical Reagent Beijing Co., Ltd., Beijing, China. In a typical procedure for the synthesis of NiSi-PMs, 0.4420 g of TEOS and 64 mL of deionized (DI) water were added to a conical flask, then 0.1291 g of NiCl_2_·6H_2_O was added to the conical flask and dissolved completely. In total, 0.8 g of urea was added to the solution followed by vigorous stirring for 0.5 h. The resultant solution was transferred to a Teflon-lined stainless steel autoclave with a capacity of 100 mL. The autoclave was sealed and heated to 210 °C and kept in an isothermal state for 12 h, then cooled down to room temperature. After being washed with DI water, the solid product was collected and finally dried at 75 °C for 12 h.

### 2.2. Preparation of Pd/NiSi-PMs Catalyst

The preparation of the catalyst included the following steps: In total, 0.5 g of NiSi-PMs was added into the solution containing 40 mL of ethanol and 60 mL of DI water in a three-neck flask equipped with a condenser under constant magnetic stirring. The flask was heated in an oil bath, heating the slurry to the boiling state and this was maintained for 1.0 h. Then, the slurry was cooled to room temperature followed by a rapid injection of 2.34 mL of PdCl_2_ solution (0.02 mol L^−1^), and kept under vigorous stirring for 1.0 h. Then, 1.88 mL of ascorbic acid solution (0.1 mol L^−1^) was dropwise added to the slurry and stirring was maintained for 0.5 h. After, the system was separated by centrifugation. The solid was dried at 75 °C for 24.0 h, and finally collected for catalytic evaluation. For comparison, three other supported Pd catalysts using activated carbon, SiO_2_, and Al_2_O_3_ as the supports were also prepared by the same procedure.

### 2.3. Characterization

X-ray diffraction (XRD) patterns were recorded on a X’Pert³ Powder X-ray powder diffractometer (PANalytical, Almelo, the Netherlands) operated at an accelerating voltage of 40 kV and an emission current of 40 mA with Cu Kα radiation. Scanning electron microscope (SEM) measurements were carried out on a JSM 6700F field emission scanning electron microscope (JEOL, Tokyo, Japan). Transmission electron microscope (TEM) measurements were carried out on a JEM–2100PLUS high resolution transmission electron microscope (JEOL, Tokyo, Japan). Nitrogen adsorption-desorption isotherms were measured using a SSA−4200 surface area and porosity analyzer (Builder Electronic Technology, Beijing, China) and the specific surface areas were calculated using the Brunauer–Emmett–Teller (BET) equation. X-ray photoelectron spectroscopy (XPS) spectra were recorded on a Thermo Scientific Escalab 250Xi spectrometer (Thermo Fisher Scientific, Waltham, MA, USA). The elemental content of the samples was determined by inductively coupled plasma analysis (ICP) on an Agilent 720 apparatus (Agilent, Santa Clara, CA, USA). H_2_ temperature-programmed reduction (H_2_-TPR) and temperature-programmed desorption (TPD) measurements were carried out on a PCA-1200 chemical adsorption instrument (Builder Electronic Technology, Beijing, China). In a typical H_2_-TPR experiment, 150 mg of catalyst was pretreated in an argon flow at 200 °C for 1.0 h. After pretreatment, the sample was cooled to room temperature. Until the baseline was stable, the TPR analysis was carried out in a 10% H_2_-Ar flow at a heating rate of 10 °C min^−1^ to 800 °C. In a typical H_2_-TPD experiment, 150 mg of catalyst was pretreated in an argon flow at 200 °C for 1.0 h. Then, the sample was cooled to room temperature and treated with 10% H_2_-Ar flow for 1.0 h. Until the baseline was stable, the desorption was carried out in flowing argon at a heating rate of 10 °C min^−1^ to 800 °C.

### 2.4. Evaluation of Catalytic Activity

In a typical procedure for the hydrogenation of styrene, 16.0 mL of ethanol and 10.0 mg of catalyst were added into a Schlenk tube, and then the Schlenk tube was purged 3 times with H_2_ after the addition of 5 mmol of styrene. The reaction was carried out under stirring at 30 °C in H_2_ atmosphere using a balloon. Reaction products were quantified by an SP-6890 gas chromatograph (Rainbow Chemical Instrument, Tengzhou, China) equipped with a flame ionization detector using a AT.FFAP capillary column.

## 3. Results and Discussion

### 3.1. Synthesis of NiSi-PMs

**Figure 1:**
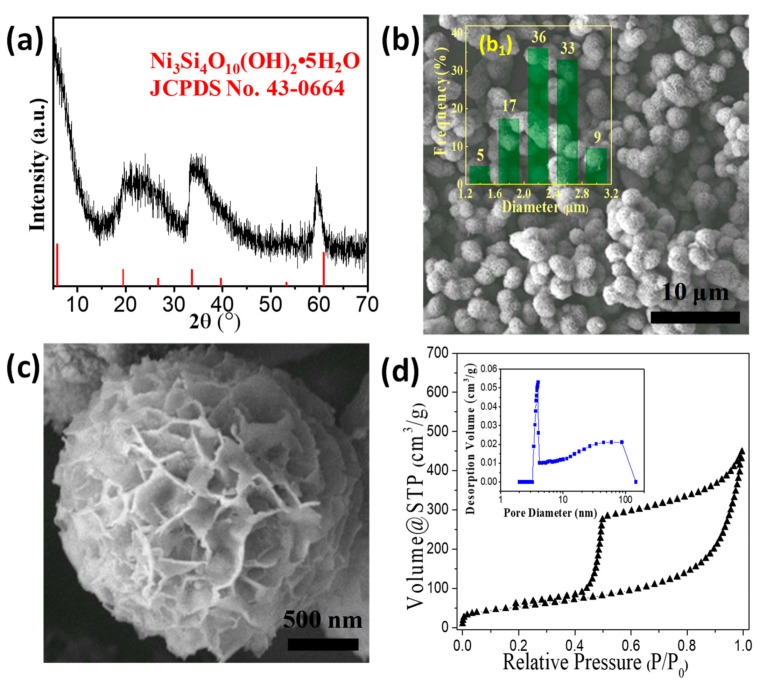
XRD pattern (**a**), SEM images (**b**,**c**), size distribution histogram (b_1_), nitrogen adsorption-desorption isotherms, and corresponding pore diameter distribution profile (**d**) of the NiSi-PMs hydrothermally synthesized at 210 °C for 12.0 h.

Figure 1 shows the XRD pattern, SEM images, and size distribution of the products hydrothermally synthesized at 210 °C for 12.0 h. The reflections in the XRD pattern can be well indexed to the hexagonal Ni_3_Si_4_O_10_(OH)_2_·5H_2_O (JCPDS No. 43-0664, a = 5.255 Å, c = 14.82 Å). Figure 1b shows that the as-synthesized product presents a uniform morphology of a porous microsphere, and the statistical data (Figure 1b_1_) demonstrates that the NiSi-PMs have a diameter of 1.2 to 3.2 μm. The magnified SEM image (Figure 1c) indicates the porous microspheres are assembled by nanosheets. The textural properties of the NiSi-PMs were evaluated based on the N_2_ adsorption-desorption experiments. The N_2_ isotherms and pore size distribution curves are shown in Figure 1d. The isotherms can be assigned to the type IV adsorption isotherms. There is a distinct hysteresis in the range from 0.4 to 1.0 of P/P_0_, which indicates the presence of mesopores. Additionally, type H2 hysteresis loops imply the ink-bottle-like pore shape. The BET surface area and pore volume of the NiSi-PMs are 196.2 m^2^ g^−1^ and 0.70 cm^3^ g^−1^, respectively. The corresponding BJH pore size distribution curve shows that a sharp peak centering around 3.9 nm exists within the pore size distribution. Combined with the SEM image (Figure 1c), it can be considered that these dominant mesopores formed in the nanosheets within the microspheres, and the assembly of the nanosheets produced piled pores with a wide pore size distribution, resulting in a much larger pore volume compared with Ni_3_Si_2_O_5_(OH)_4_ nanotubes [20,21] and core-shell spheres [22,23].

**Figure 2:**
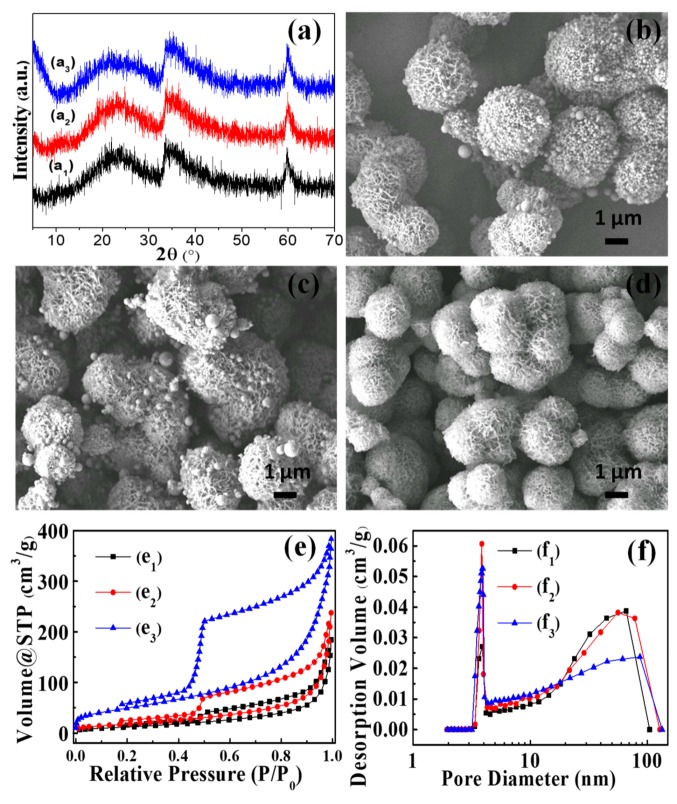
XRD patterns (**a**), SEM images (**b**–**d**), nitrogen adsorption-desorption isotherms (**e**), and pore diameter distribution profiles (**f**) of the products hydrothermally synthesized at 120 °C (a_1_, (**b**), e_1_, f_1_), 150 °C (a_2_, (**c**), e_2_, f_2_), and 180 °C (a_3_, (**d**), e_3_, f_3_) for 12.0 h.

The effect of the hydrothermal temperature was investigated to better understand the formation of the NiSi-PMs. Figure 2 shows that the hydrothermal temperature has an appreciable effect on the product morphology and pore structure. With the hydrothermal temperature ranging from 120 to 180 °C, all the products are Ni_3_Si_4_O_10_(OH)_2_ with a similar crystallinity (Figure 2a). Notably, however, the morphology of the products varied significantly with the increase of the hydrothermal temperature. When hydrothermally treated at 120 or 150 °C, the products exhibited the morphology of microspheres, and at the same time, a large number of small spherical particles were found in the piled pores and on the surface of the microspheres. These small spherical particles are assumed as silica formed by the hydrolysis of TEOS. When the hydrothermal temperature was increased to 180 °C, the small spherical particles disappeared and the products exhibited a similar morphology to the products obtained at 210 °C (Figure 1b). The N_2_ isotherms (Figure 2e) show that the BET surface areas increase significantly with the increase of the hydrothermal temperature. The BET surface areas and pore volumes are 47.6 m^2^ g^−1^ and 0.29 cm^3^ g^−1^, 68.5 m^2^ g^−1^ and 0.37 cm^3^ g^−1^, and 183.8 m^2^ g^−1^ and 0.60 cm^3^ g^−1^, for the products obtained at 120, 150, and 180 °C, respectively. The pore size distribution curves (Figure 2f) indicate that all the products have dominant mesopores centered at 3.9 nm. Meanwhile, wide pore size distributions in the range from 20 to 100 nm were observed for all the products, which are attributed to the piled pores as aforementioned.

During hydrothermal treatment, decomposition of urea in aqueous solution leads to the release of CO_2_ and NH_3_ into the system, and the decomposition rate is significantly affected by temperature. NH_3_ can easily dissolve into water, generating NH_4_^+^ and OH^−^ ions. In this basic aqueous solution, TEOS reacts with Ni^2+^ and OH^−^ ions to form Ni_3_Si_4_O_10_(OH)_2_. With the gradual decomposition of urea, OH^−^ ions are constantly released. Base-catalyzed hydrolysis of TEOS takes place, leading to the formation of SiO_2_. Therefore, there is SiO_2_ coexisting with Ni_3_Si_4_O_10_(OH)_2_ in the products under the condition of a low hydrothermal temperature. As the reaction approaches completion, the concentration of OH^−^ ions is thereby increased. Then, the SiO_2_ formed through the hydrolysis of TEOS is etched under this elevated alkaline condition, while Ni_3_Si_4_O_10_(OH)_2_ can be further generated by the reaction of SiO_2_, Ni^2+^, and OH^−^. On the other hand, fast decomposition of urea leads to the presence of high concentrations of CO_2_ and OH^−^ at high hydrothermal temperatures. So, the reaction is significantly accelerated, and at the same time, a large amount of CO_2_ bubbles stay in the solution, which can be heterogeneous nucleation centers according to the previous reports [24,25]. Ni_3_Si_4_O_10_(OH)_2_ tends to aggregate together around the CO_2_ bubbles and self-assemble into larger particles, resulting in the porous structure of the final products. So, the size of NiSi-PMs is much larger than that of the products obtained in the ammonia chloride and NH_3_·H_2_O system [26,27].

### 3.2. Performance of the Pd/NiSi-PMs Catalyst for the Hydrogenation of Styrene

**Figure 3:**
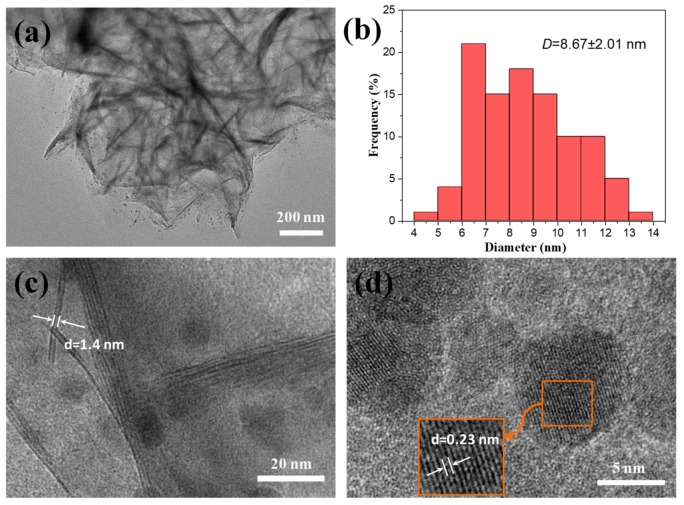
TEM images (**a**,**c**,**d**) and the size distribution of Pd nanoparticles (**b**) of the Pd/NiSi-PMs catalyst.

**Figure 4:**
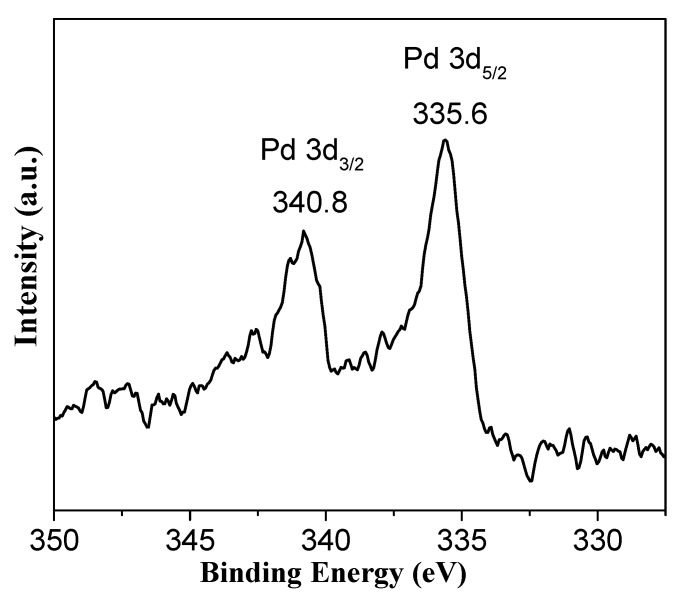
Pd 3d XPS spectrum of the Pd/NiSi-PMs catalyst.

The TEM images of Pd/NiSi-PMs are shown in Figure 3. It is confirmed that the porous microspheres are composed of nanosheets. Pd nanoparticles can be seen supporting in the NiSi-PMs with good dispersion, and Figure 3a shows that most of them are distributed in the outer edge of the microspheres. The statistical data for the size distribution of the supported Pd particles are shown in Figure 3b, and the particle size is 8.7 ± 2.0 nm. In Figure 3c, a layered structure can be found and the inter-lamellar spacing is 1.4 nm, which corresponds to the (001) plane of Ni_3_Si_4_O_10_(OH)_2_. Figure 3d shows that the Pd nanoparticles are highly crystalline, and the interplanar spacing is 0.23 nm, which is assigned to the (111) plane of the cubic Pd structure. The chemical state of Pd in the Pd/NiSi-PMs catalyst was examined by XPS measurement. The Pd 3d spectrum is shown in Figure 4. The binding energies of Pd 3d_5/2_ and Pd 3d_3/2_ were determined to be 335.6 eV and 340.8 eV, respectively, which are characteristic of metallic Pd.

**Figure 5:**
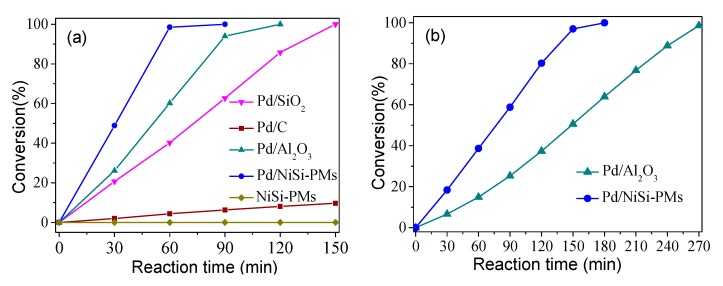
Performances of different catalysts for styrene hydrogenation. (**a**): 5 mmol styrene, (**b**): 10 mmol styrene.

The catalytic property of the synthesized Pd/NiSi-PMs for the hydrogenation of styrene was evaluated at 30 °C in an H_2_ atmosphere. For comparison, several other supported Pd catalysts using the conventional supports, such as activated carbon, SiO_2_, and Al_2_O_3_, were also prepared via the same method and employed for the same reaction. The actual amounts of Pd loading on the supports were analyzed by ICP characterization, and the weight percentages of Pd in Pd/C, Pd/Al_2_O_3_, Pd/SiO_2_, and Pd/NiSi-PMs were 1.03%, 0.95%, 0.92%, and 0.96%, respectively. The weight percentage of Ni in Pd/NiSi-PMs was 32.4%, which was very close to the theoretical value of Ni in Ni_3_Si_4_O_10_(OH)_2_·5H_2_O (30.8%). Figure 5 shows styrene conversions on these catalysts as a function of the reaction time. When the catalytic reactions were carried out with 5 mmol styrene in 16 mL of ethanol as the solvent (Figure 5a), the styrene conversions were 2.0%, 20.7%, 26.1%, and 48.9% after a 30 min reaction on Pd/C, Pd/SiO_2_, Pd/Al_2_O_3_, and Pd/NiSi-PMs, respectively, and further increased to 4.4%, 40.2%, 60.2%, and 98.5% when the reaction time was prolonged to 60 min. The selectivity to ethylbenzene was 100% on all of the catalysts. The catalytic performance of the NiSi-PMs support was also evaluated and is shown in Figure 5a. Without Pd loading, NiSi-PMs were inactive for the hydrogenation of styrene. It can be found that the styrene conversion increases nearly linearly with the increase of the reaction time for each catalyst. The turnover frequencies (TOFs, moles of substrate converted per mole of Pd per hour) on every catalyst were calculated according to the linear fitting results of the conversions in the first 60 min, and the results are 230, 2152, 3118, and 5234 h^−1^ for Pd/C, Pd/SiO_2_, Pd/Al_2_O_3_, and Pd/NiSi-PMs, respectively. When the concentration of the substrate was increased to 10 mmol styrene in 16 mL ethanol, the catalytic performances of Pd/Al_2_O_3_ and Pd/NiSi-PMs were obtained as shown in Figure 5b. The TOF values were 1993 and 4173 h^−1^ according to the linear fitting results of the conversions in the first 150 min, displaying an obvious decline in comparison with the TOF values under the lower substrate concentration, which is consistent with the previously reported results [28].

**Figure 6:**
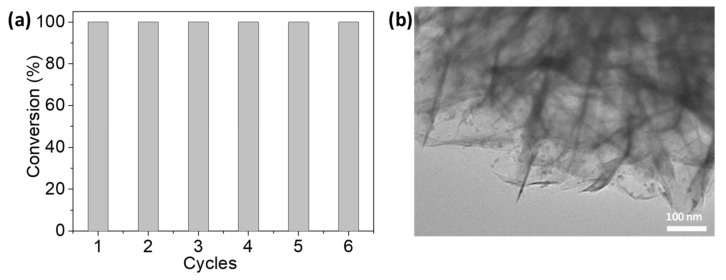
The recyclability (**a**) of Pd/NiSi-PMs for the hydrogenation of styrene, and the TEM image (**b**) of the used catalyst after the sixth cycle.

To give better insight into the catalytic property of the Pd/NiSi-PMs catalyst, comparison with the results in the literature using other catalysts was made, as shown in Table 1. As shown, various materials have been used as the support of Pd catalysts, including MOFs, organic polymers, oxides, hydroxide, and other composites. The TOF value on Pd/NiSi-PMs is comparatively higher than those on most of the referenced catalysts, and moreover, this catalyst is competitive in view of the simple preparation process and the use of relatively cheap raw materials compared with the complex composite or rare earth-containing compounds. So, the as-obtained NiSi-PM is a promising candidate as the support for noble metal catalysts. The durability of Pd/NiSi-PMs was evaluated by investigating its recycling performance. After completion of a catalytic reaction, the catalyst was removed from the product by centrifugation and then reused in another catalytic cycle. This procedure was repeated for six consecutive cycles without any discernible loss of activity as shown in Figure 6a. TEM and ICP characterizations of the used Pd/NiSi-PMs catalyst were carried out after the sixth cycle. The size of Pd nanoparticles did not change apparently, as shown in Figure 6b, and the weight percentage of Pd in the catalyst was 0.92%, indicating that Pd/NiSi-PMs have good recyclability and stability as heterogeneous catalyst.

### 3.3. H_2_-TPR and H_2_-TPD Analysis of the Catalysts

**Figure 7:**
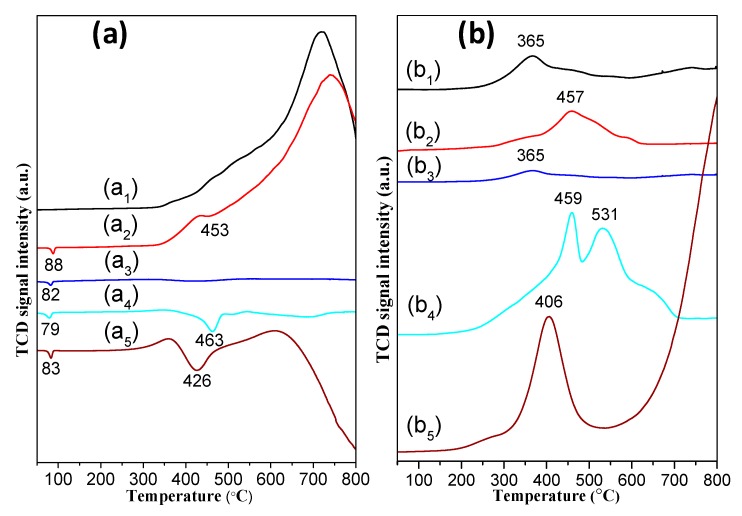
H_2_-TPR (**a**) and H_2_-TPD (**b**) profiles of NiSi-PMs (a_1_, b_1_), Pd/NiSi-PMs (a_2_, b_2_), Pd/SiO_2_ (a_3_, b_3_), Pd/Al_2_O_3_ (a_4_, b_4_), and Pd/C (a_5_, b_5_).

The hydrogenation of alkene to alkane with H_2_ has been described by the Horiuti–Polanyi mechanism [37]. The first step is hydrogen dissociation on the metal surface. Then, the adsorption of alkene is followed by the sequential addition of hydrogen atoms to alkene. The formation and desorption of the alkane is the final step. Generally, the conversion of alkene is considered to be closely connected to the adsorption state of hydrogen atoms on the metal surface [38]. H_2_-TPR and H_2_-TPD were performed to explore the possible reason for the significant differences in catalytic property among the Pd supported catalysts with different support. Figure 7a shows the H_2_-TPR profiles of NiSi-PMs and the Pd supported catalysts. There is a broad reduction peak starting at 300 °C with a *T_max_* at 721 °C in the TPR profile of NiSi-PMs (Figure 7a_1_) due to reduction of the nickel species. After Pd was loaded, as shown in Figure 7a_2_, the main reduction peak is very similar to that of the NiSi-PMs support, indicating that the valence state of nickel and the structure of NiSi-PMs were unchanged during the preparation of the catalyst. On the other hand, a new negative peak at 88 °C appears in the profile of Pd/NiSi-PMs. In the profiles of the other Pd supported catalysts, negative peaks can also be observed at 82, 79, and 83 °C for Pd/SiO_2_, Pd/Al_2_O_3_, and Pd/C, respectively. It has been reported [39,40] that the interstitial solid solution β-PdH_x_ can be formed in Pd supported catalysts with the absorption of hydrogen into the sub-surfaces of metallic Pd. Thus, the negative peaks below 90 °C correspond to the decomposition of β-PdH_x_. Moreover, Pd/NiSi-PMs, Pd/Al_2_O_3_, and Pd/C show the presence of another negative peak at 453, 463, and 426 °C, respectively. They may be ascribed to the desorption of the chemisorbed hydrogen on palladium and the spillover hydrogen, which was activated on metallic Pd to the support. Unlike the other three catalysts, Pd/C shows a significant negative signal from about 620 °C in the H_2_-TPR profile, indicating that strong adsorption centers for hydrogen exist in the Pd/C catalyst and their amount is apparently much higher than the adsorption centers corresponding to the negative peak at 426 °C. The adsorption of hydrogen on NiSi-PMs and the Pd supported catalysts was further investigated by H_2_-TPD as shown in Figure 7b. There is a desorption peak with a *T_max_* at 365 °C in the H_2_-TPD profile of NiSi-PMs as shown in Figure 7b_1_, representing a certain adsorption capacity for hydrogen. Note that though crystalline water and constitution water is released from NiSi-PMs under high temperatures, the peak in the H_2_-TPD profile was only ascribed to the desorption of hydrogen, which had been adsorbed at room temperature, because water was eliminated before the flow entered into the detector, and there was no other species coming out. With Pd loading, the Pd/NiSi-PMs shows a much larger adsorption capacity (Figure 7b_2_). Moreover, the *T_max_* value in the profile of Pd/NiSi-PMs increased to 457 °C, indicating that the chemisorption state of hydrogen on the catalyst is different to that on the support. For the Pd supported catalysts, it is clear that the adsorption capacity for hydrogen is in the order of Pd/C > Pd/Al_2_O_3_ > Pd/NiSi-PMs > Pd/SiO_2_. The profile of Pd/C demonstrates the presences of two kinds of adsorption centers just as the above H_2_-TPR analysis. Two peaks centered at 459 and 531 °C, and a shoulder peak between 600 and 700 °C are observed in the profile of Pd/Al_2_O_3_, indicating complex adsorption sites. There is only one small desorption peak at 365 °C in the profile of Pd/SiO_2_, representing the weak adsorption ability for hydrogen. As compared with Pd/SiO_2_, the adsorption strength and amount on Pd/NiSi-PMs are much higher, indicating that the existence of nickel enhanced the adsorption ability for hydrogen. The decomposition of β-PdH_x_ gives signals in the H_2_-TPR profiles as described above, but is not reflected in the H_2_-TPD profiles, which shows the β-PdH_x_ has decomposed at room temperature in the argon flow.

The above results reveal that there are multiple adsorption states of hydrogen atoms on these supported Pd catalysts. It is obvious that strong adsorption centers are dominant in Pd/C. Although weakly and moderately bound hydrogen existed in Pd/C, which can be respectively assigned to the peaks below 100 °C and at 300 to 500 °C in the H_2_-TPR and H_2_-TPD profiles, the hydrogen dissolved in the solution under the reaction condition was probably preferentially converted to strongly bound hydrogen, which seems not to be the effective hydrogen species for styrene hydrogenation in the liquid phase at room temperature, according to the poor catalytic performance of Pd/C as shown in Figure 5. For comparison, Pd/SiO_2_ provides only weak and moderate adsorption centers, and shows a much lower adsorption capacity for hydrogen, yet its catalytic activity is higher than Pd/C. Pd/NiSi-PMs shows more weakly and moderately bound hydrogen than Pd/SiO_2_, and meanwhile, higher catalytic activity was obtained. More complicated adsorption states of hydrogen are found on Pd/Al_2_O_3_, which shows a higher capacity for moderately bound hydrogen than Pd/NiSi-PMs, but also provides stronger adsorption centers as revealed by H_2_-TPD measurement, unfavorable for the hydrogenation reaction. The NiSi-PMs support shows an adsorption ability for hydrogen as revealed by H_2_-TPD, however, it is inactive for the reaction. Without Pd loading, it is probably not dissociative adsorption of hydrogen on the NiSi-PMs support, which is necessary for the hydrogenation of alkenes.

## 4. Conclusions

Nickel phyllosilicate Ni_3_Si_4_O_10_(OH)_2_ with a porous microspherical structure can be fabricated via a urea-assisted hydrothermal method using NiCl_2_, TEOS, and urea as the raw materials. The decomposition of urea, which can be controlled by hydrothermal conditions, directly affected the concentration of OH^−^ ions and CO_2_ in the system. Ni_3_Si_4_O_10_(OH)_2_ was formed rapidly by the reaction of OH^−^, Ni^2+^ and TEOS. Meanwhile, a high concentration of OH^−^ ions promotes the hydrolysis of TEOS, leading to the formation of SiO_2_, which can be etched under elevated alkaline conditions. CO_2_ bubbles in the solution provide heterogeneous nucleation centers for the formation of the porous microspherical structure. The obtained Pd/NiSi-PMs catalyst exhibited a TOF of 5234 h^−1^ for the hydrogenation of styrene. The high catalytic activity was probably attributed to the unique pore structure and the appropriate adsorption property for hydrogen. The present Ni_3_Si_4_O_10_(OH)_2_ supported Pd catalyst has the advantages of good catalytic activity and recyclability, and ease of catalyst preparation. Therefore, it is a promising and competitive catalyst from a practical point of view, and is expected to have a broad range of utility for the industrial application.

## Figures and Tables

**Table 1:** Comparison of Pd/NiSi-PMs with other catalysts on the catalytic activity for styrene hydrogenation.

Catalyst	Pd Loading (wt.%)	Size of Pd Nanoparticles (nm)	H_2_ Pressure (atm)	Solvent	TOF (h^−1^)	Reference
Pd/ZIF-8	1	NA ^a^	1 ^b^	None	307	[29]
Pd/Tm-MOF	1	NA ^a^	1 ^b^	None	703	[29]
Pd/C	10	NA ^a^	40.8 ^b^	Ethyl acetate	537	[30]
Pd/PEG	3.75	5	1 ^b^	Ethanol	660	[28]
polymer anchored palladium	12.1	NA ^a^	1 ^b^	DMF	766	[31]
Fe_3_O_4_-NC-PZS-Pd	3.6	3.5 ± 1.5	1 ^b^	Ethanol	1792	[32]
Pd/h-WO_3_	1	6	1 ^b^	Ethanol	3050	[33]
Pd/PIL-Br	10	3.3	10 ^b^	Methanol	4800	[34]
Pd/TiO	1	10	1 ^c^	Ethanol	4838	[35]
Pd/NiSi-PMs	1	8.7 ± 2.0	1 ^b^	Ethanol	5234	This work
Pd/Gd(OH)_3_	0.95	2-3	1 ^c^	Ethanol	6159	[36]

^a^ NA = not available; ^b^ static hydrogen atmosphere. ^c^ H_2_ flow rate of 40 mL min^−1^.
